# Dark-State-Mediated Efficient Energy Trapping in a Model GFP Chromophore

**DOI:** 10.1021/jacs.5c22023

**Published:** 2026-04-17

**Authors:** Elisabeth Gruber, Lars H. Andersen, Laurence H. Stanley, Jan R. R. Verlet, Ivan S. Avdonin, Anastasia V. Bochenkova

**Affiliations:** † Department of Physics and Astronomy, 27255 Aarhus University, Aarhus C 8000, Denmark; ‡ Department of Chemistry, 3057 Durham University, Durham DH1 3LE, United Kingdom; § Department of Chemistry, 64935 Lomonosov Moscow State University, Moscow 119991, Russia

## Abstract

The functional properties of photoactive proteins are governed by the interplay between bright and dark excited states. While the bright states are well-studied, the dark states, which are fundamental to photostability and light harvesting, are notoriously difficult to characterize. Here, we report the direct observation and full characterization of an optically dark, low-lying singlet excited state in the isolated anion of the meta green fluorescent protein (GFP) chromophore. Using a combination of ultrafast time-resolved action absorption and photoelectron spectroscopy, we have captured the formation of this state in 100 fs and measured its remarkably long lifetime of 94 ps. We unambiguously assign its charge-transfer character and reveal the precise trapping mechanism through high-level *ab initio* calculations. Our findings uncover a photoprotective mechanism in biomolecular anions, where ultrafast internal conversion quenches electron emission, stabilizing long-lived electronic excitation even when the energy exceeds the electron detachment threshold.

## Graphical abstract

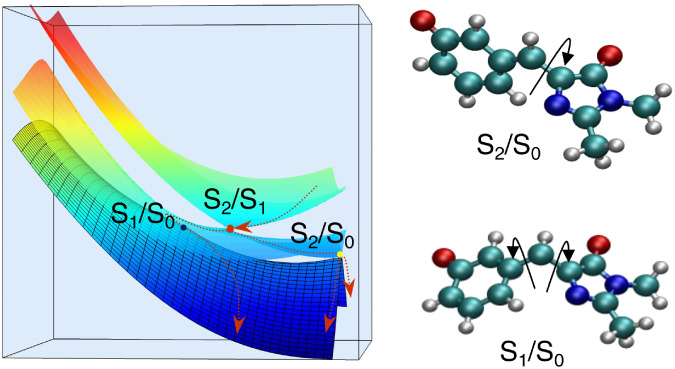

## Introduction

At first sight, one might think that optically dark states (i.e., electronic states having a small dipole coupling with the electronic ground state, or dark because of symmetry reasons) are of little interest. They do, however, play an important role in the photophysics of chromophores as they may be populated through conical intersections after photoexcitation to higher lying bright electronic states. Upon internal conversion, they may thus act as “trapping states” with long lifetimes and little fluorescence. In photosynthetic complexes, symmetry-forbidden dark states are relevant in light-harvesting and for photoprotection by carotenoids.^1–3^ When excited to the bright S_2_ state, the carotenoid chromophore relaxes within 50–300 fs to the optically inactive S_1_ state, which is then involved in the pigment–pigment energy transfer process. In carotenoids containing carbonyls, it is found that the lifetime of the dark charge-transfer states is strongly solvent dependent.^4^

Chromophores featuring long-lived dark excited states are of considerable interest for their potential in advanced technologies. They might be applicable in biomimetic electronic and memory devices, where there is a need to switch between light emitting and nonemitting states.^5^ The utility of these states also extends to sensing and photoprotection. Their long lifetime may enable their use as “dark donors” in Förster resonance energy transfer, providing a background-free signal for biosensing.^6^ Simultaneously, their capacity to absorb harmful high-energy photons via a higher-lying bright state and to dissipate the energy as heat from a stable dark state makes them highly efficient energy sinks, offering a strategy for enhancing the durability of polymers and synthetic materials.^7^ Given the above, it is not surprising that there has been a desire to develop photoactive proteins with engineered dark states. Specifically, dark charge-transfer states have been studied in the chromophores of the photoactive yellow protein (PYP)^8^ and the Green Fluorescent Protein (GFP)^9–11^ through their meta-derivatives. Dark states also play an important role in the function of visual pigments. Recent work demonstrates that the mixing of the dark and optically allowed states modulates the quantum efficiency of ultrafast photoisomerization.^12^

Solvation can strongly influence dark states, underscoring the need to understand their intrinsic spectroscopy and dynamics. Gas-phase spectroscopy uniquely enables such studies by isolating the intrinsic properties. Yet, the optically forbidden nature of dark states renders their investigation experimentally challenging, especially in the gas phase where ion densities are low. To overcome this problem we apply action-absorption spectroscopy, developed for studies of chromophore ions at ion-storage rings both in terms of spectroscopy^13,14^ and lifetimes^15,16^ alongside photoelectron spectroscopy^17,18^ and high-level quantum chemical calculations.^19–21^

Here we focus on the chromophore of the GFP. This protein, with 238 amino acids, has a rigid 11-stranded β-barrel structure with a 4-(*p*-hydroxybenzylidene)-5-imidazolinone (pHBI) chromophore at the center, well protected from external solvents. The protein is widely used for imaging purposes in biological samples due to its fluorescent properties.^22^ Numerous studies of GFP-model chromophores have been conducted over the past almost 20 years. In particular, a dimethyl derivative of pHBI called para-HBDI, 4-hydroxybenzylidene-2,3-dimethyl-imidazolinone, is used as a model for the chromophore of the protein. For this molecule, the absorption spectrum within the visible range is dominated by the intense S_0_ → S_1_ transition at ∼480 nm^13^ (see Figure 1), with a band origin, determined by cryogenic spectroscopy, at 481.5 nm.^23^ Above the electron-detachment threshold, electron emission proceeds through autodetachment of vibrational resonances (VRs), whereas below the threshold there may appear a significant contribution from sequential multiphoton absorption in action-absorption spectra mediated by the strong S_0_ → S_1_ transition in para-HBDI.^11,13,20,24^ The separation between one and multiphoton contributions is conveniently done by the use of fast fs-laser pulses.^25^

**Figure 1:**
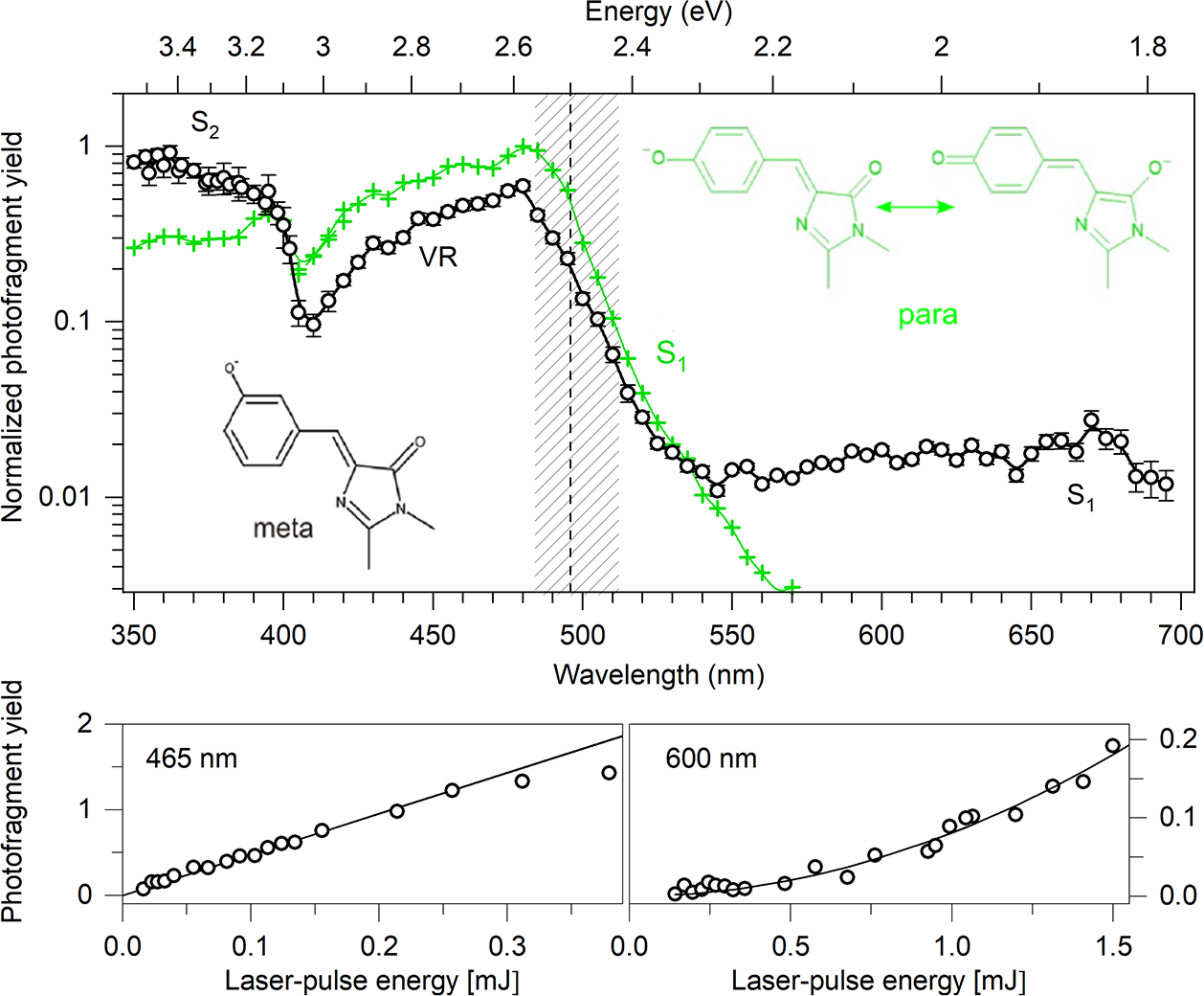
Action-absorption spectrum of meta-HBDI (black data points) in comparison to para-HBDI (green data points). Notice the logarithmic vertical axis. The vertical line shows the electron-detachment threshold for the meta chromophore. Between 350 and 500 nm prompt action is detected after single-photon absorption (photofragment yield increases linearly with the laser-pulse energy, presented here for 465 nm in the lower graph) and associated with excitation into S_2_ and vibrationally resonant (VR) photodetachment out of the dipole-bound state. The weak absorption between 550 and 700 nm for meta-HBDI is attributed to excitation of the dark S_1_ state. Sequential absorption of two photons is necessary to cause fragmentation (photofragment yield increases quadratically with the laser-pulse energy, presented here for 600 nm in the lower graph). Data in the region 550 and 700 nm is normalized according to the quadratic laser-power dependence.

Moving the oxygen atom on the phenolate ring from the para to the meta position (see Figure 1) significantly alters the low-energy part of the HBDI photoabsorption spectrum, which is relevant to its photochemistry.^11^ The absence of valence resonance structures of the meta-HBDI anion in the electronic ground state causes an effective decoupling of the isoelectronic π-systems of the phenolate and imidazolinone rings. Calculations of the relevant π and π* orbitals reveal that the S_0_ → S_1_ transition has a significant charge-transfer character, implying that this S_1_ state is strongly influenced by the solvation of a polar solvent (e.g., water). It is predicted to lie at the far red edge of the visible spectrum (∼700 nm) with a very low oscillator strength.^11^

This work offers direct spectroscopic access to the dark S_1_ state of meta-HBDI, revealing its lifetime and energy. The photoresponse of the anion has been studied in the spectral region from 350 to 700 nm in the gas phase. Through a combination of action-absorption and photoelectron spectroscopy, we experimentally confirm the existence of this low-lying dark state, previously predicted theoretically, and characterize its charge-transfer nature by monitoring the blue shift induced upon complexation with betaine, a molecule possessing a large dipole moment. To elucidate the underlying energy flow, we map the excited-state dynamics using pump–probe techniques supported by *ab initio* calculations. Our combined experimental and theoretical approach provides a comprehensive framework for understanding the competition between internal conversion and electron detachment, particularly when the anion is excited above its photodetachment threshold.

## Results and Discussion

### Nature and Energy Landscape of Excited States

Figure 1 shows the action-absorption spectrum of meta-HBDI in the spectral region from 350 to 700 nm, covering transitions from the electronic ground state S_0_ into the first excited state S_1_ (weak absorption between 550 and 700 nm) and into the second excited state S_2_ (below 400 nm). The vertical electron detachment energy (VDE) for meta-HBDI was previously determined to be in the range of 2.40–2.54 eV ± 0.1 eV.^11^ The significant spread was due to inhomogeneous broadening caused by internal rotation about the single C–C bridge bond in the electronic ground state. Indeed, MP2/(aug)-cc-pVTZ calculations reveal two rotamers in meta-HBDI, separated by an energy of 0.05 eV with an interconversion barrier of 0.46 eV (see Figure S1). While these rotamers are spectroscopically indistinguishable at their equilibrium planar geometries, rotation along the interconversion coordinate lowers the VDE, with a maximum reduction of 0.2 eV at the transition state. Here, using photoelectron spectroscopy, we refine the experimental VDE and also find the adiabatic detachment energy to be 2.63 and 2.30 ± 0.05 eV, respectively.

The broad and intense absorption observed between 400 and 500 nm—near and above the electron-detachment threshold—arises from two main processes: direct electron detachment, and excitation of vibrational resonances associated with a nonvalence, electronically dipole-bound state.^11^ States with little vibrational excitation are truly bound and are observed experimentally through sequential two-photon absorption. In contrast, excess vibrational energy in the radical core can promote these states into the continuum, where they manifest as autodetaching resonances.^18,26,27^ In meta-HBDI, direct transitions from S_0_ to these states are spectrally distinct from transitions to the valence excited states: S_1_ in the near-IR, which is electronically bound, and S_2_ in the UV, which is embedded in the continuum and is therefore metastable against electron emission.

The charge-transfer nature of the S_0_ → S_1_ transition was revealed by zwitterion tag action spectroscopy (ZITA spectroscopy).^28^ The excitation of a charge-transfer (CT) transition significantly redistributes electron density within the chromophore. This redistribution alters the Coulombic interaction with any nearby dipole, making CT transitions exceptionally sensitive to their electrostatic environment, including solvent interactions. The different interaction strength between the ion and the dipole in the ground and excited states leads to a blueshift of the absorption band. Thereby, the extent of the shift is a measure of the CT degree. With a 11.9 D dipole moment arising from its quaternary ammonium and carboxylate termini,^29^ the zwitterion betaine is an excellent probe molecule for ZITA spectroscopy.^28,30,31^

The absorption of the betaine-meta-HBDI complex shows a more than 100 nm blue-shift for the S_0_ → S_1_ transition energy, pointing to a strong CT character (see Figure 2). In contrast, only a small blue-shift is observed for the 400 nm transition into S_2_, pointing to a much lower CT character. This difference in CT character can be understood by considering the electronic structure of the meta-HBDI anion, which is best described as two isoelectronic π-systems comprising eight electrons distributed over seven orbitals. The first subsystem includes the phenolate ring (Ph^–^), which carries a negative charge in the ground state, while the second comprises the neutral imidazolinone ring and the bridging carbon atom (Im=C). These subsystems are effectively decoupled due to the absence of valence resonance structures in the meta-HBDI anion in the ground electronic state. Optical transitions in the full system may therefore be classified as either intra- or intersubsystem excitations (see Figure S2). Consistent with this picture, the S_0_ → S_1_ transition involves a significant transfer of electron density from the phenolate ring to the Im=C part. This interpretation is supported by the calculated change in the electrostatic dipole moment (Δμ = 12.5 D) and by analysis of the π and π* orbitals primarily involved in the excitation (inset, Figure 2). In contrast, the S_0_ → S_2_ transition corresponds mainly to a local redistribution of electron density within the imidazolinone ring and the bridge C=C bond. Accordingly, the computed dipole moment change is substantially smaller (Δμ = 7.0 D). For the bare anion, the moderate residual CT character in this transition arises from an admixture of a doubly excited intersubsystem configuration, which transfers charge from the highest occupied orbital of the first π-system to the lowest unoccupied orbital of the second π-system (inset, Figure 2). Further evidence for the distinct characters of the two transitions is provided by differential electron density maps (Figure S3) and by the total charges of the subsystems in the relevant electronic states (Figure S4). This behavior contrasts with that of the para-HBDI anion, where resonance delocalization of the negative charge over two oxygen atoms equalizes the two bridge bonds (inset, Figure 1) and confers a distinct electronic structure on the S_1_ state.^20^ In that case, the bright S_0_ → S_1_ excitation corresponds to electron density redistribution within an allyl-anion-like bridge moiety (Figure S4). Consistently, the computed change in dipole moment upon excitation is only moderate (Δμ = 2.0 D), reflecting the absence of significant charge transfer between distinct subsystems.

**Figure 2:**
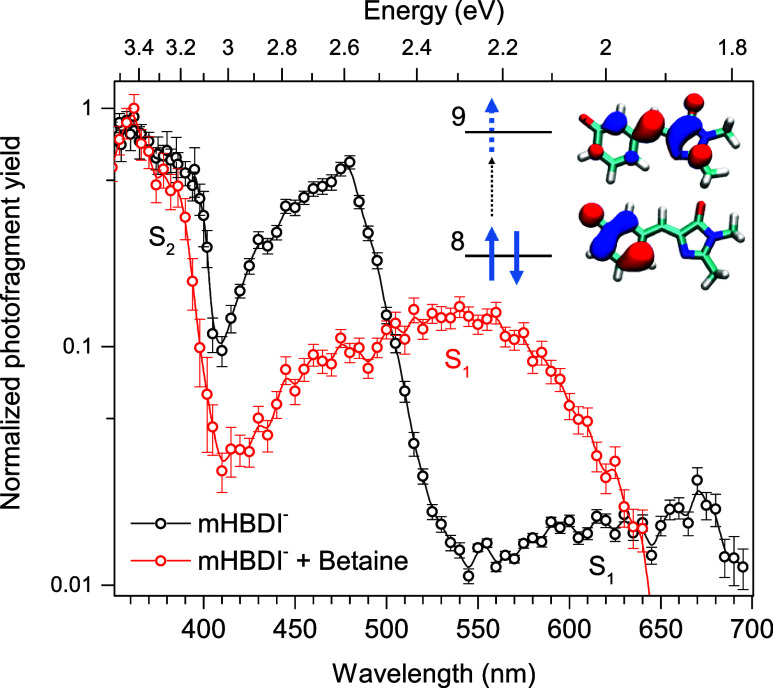
Prompt action spectrum of meta-HBDI (black data points) in comparison with meta-HBDI complexed with betaine (red data points). A strong blueshift of the S_0_–S_1_ transition energy in the meta-HBDI–betaine complex reflects a strong CT character. Calculations of the π and π* orbitals, which are primarily involved in the excitation, show that the transition is connected to charge transfer from the phenolate ring into the imidazolinone ring. Only a little blueshift for the transition into S_2_ is observed.

It is worth noting that the S_0_ → S_2_ transition to the bright, locally excited state has been previously observed for the meta-HBDI anion in solution, with an absorption band around 350–380 nm.^9^ This closely resembles the gas-phase absorption observed below 400 nm in the present work. The agreement supports the local excitation character of this transition, which exhibits limited charge-transfer character and is therefore largely insensitive to the polar solvent environment. The dark, charge-transfer transition to the S_1_ state is not amenable to direct characterization in solution due to its very low oscillator strength.

The presence of the low-lying dark state in the meta-HBDI anion is further supported by photoelectron spectroscopy and high-level *ab initio* calculations. Figure 3 shows the two-dimensional photoelectron spectrum^18,32,33^ of meta-HBDI recorded with nanosecond laser pulses with one-dimensional cuts at specific photon energies. Several distinct regions, labeled (*i*) – (*v*), are highlighted for discussion. With reference to the energy levels, calculated using multiconfiguration quasi-degenerate perturbation theory XMCQDPT2 ^34^ (see Figure 4 and the SI for computational details), these regions correspond to the following processes:

(1) The linear relation between the photon energy and the energy of the outgoing electron corresponds to direct photodetachment *hv* + S_0_ → D_0_ + e^–^. Excitation at 4.28 eV yields the most unperturbed photoelectron spectrum (see Figure 3b, lower right panel). We determine adiabatic and vertical electron detachment energies of 2.30 ± 0.05 eV and 2.63 ± 0.05 eV, respectively, which are consistent with and refine the previously measured values of 2.40–2.54 ± 0.1 eV.^11^ The calculated vertical detachment energy (D_0_) is 2.54 eV, which corresponds well to the ridge of (*i*) in the photoelectron spectrum.
(2) Similarly, region (*ii*) corresponds to direct detachment to an excited state in the neutral radical D_1*n*_. The calculated difference between D_1*n*_ and D_0_ is 1.04 eV, which matches the experimental gap.
(3) The appearance of region (*iii*) with an electron-kinetic energy higher than that of direct one-photon detachment provides evidence for a two-photon detachment process. This mechanism requires the first photon to populate an intermediate state of the anion, from which a second photon, absorbed within the same laser pulse, causes electron detachment. In the ns-laser experiments, the weak signal in region (*iii*) is assigned to photodetachment from the intermediate S_1_ state. Electrons ejected from this state are born with an adiabatic excitation energy (AEE) of 1.51 eV (see Figure 4). For a process where S_1_ is populated by internal conversion from S_2_, the sequential absorption of two 3.02 eV photons yields photoelectrons with a maximum kinetic energy of AEE­(S_1_) + *hv* – VDE­(D_0_) ∼ 2.0 eV (Figure 3b, upper left panel). The peak is significantly enhanced with 400 nm (3.1 eV) fs-laser pulses (Figure 3b, upper right panel) due to the higher probability of absorbing a second photon from the intermediate S_1_ state within the tens-of-fs pulse duration compared to the much longer 5 ns pulse. Although absorption from S_2_ could yield a peak at AEE­(S_2_) + *hv* – VDE­(D_1_) ∼ 2.0 eV, its lifetime is much shorter (see below) than that of S_1_ and comparable to the femtosecond pulse duration, rendering this pathway less probable.
(4) The region of low-energy electrons (*iv*) is due to internal conversion from S_2_ to S_1_ and further to S_0_ followed by statistical thermionic emission from the ground state^35^ or vibrational autodetachment out of the electronically bound S_1_ state.^23^ This channel may also contain a contribution from sequential absorption of multiple photons from the ns-laser pulses (duration ∼ 5 ns). Internal conversion is here faster than autodetachment out of S_2_. This resonance state is of a Feshbach type with respect to the open D_0_ continuum,^11^ and hence electronic autodetachment out of this state is a two-electron process, which becomes slower than the sub-ps nuclear photoresponse in this region.^36,37^

(5) Finally, in region (*v*) between the 3.6 and 3.9 eV photon energy there is a slight broadening in the signal, which may be caused by the opening of the resonant channel *hv* + S_0_ → S_3_ → D_0_ + e^–^ (Figures 3a and 3b, lower left panel). The calculated position of S_3_ supports this assignment (see Figure 4). This state is a shape resonance with respect to the D_0_ continuum,^11^ and one-electron autodetachment dominates the signal in region (*v*).^21,38^

**Figure 3:**
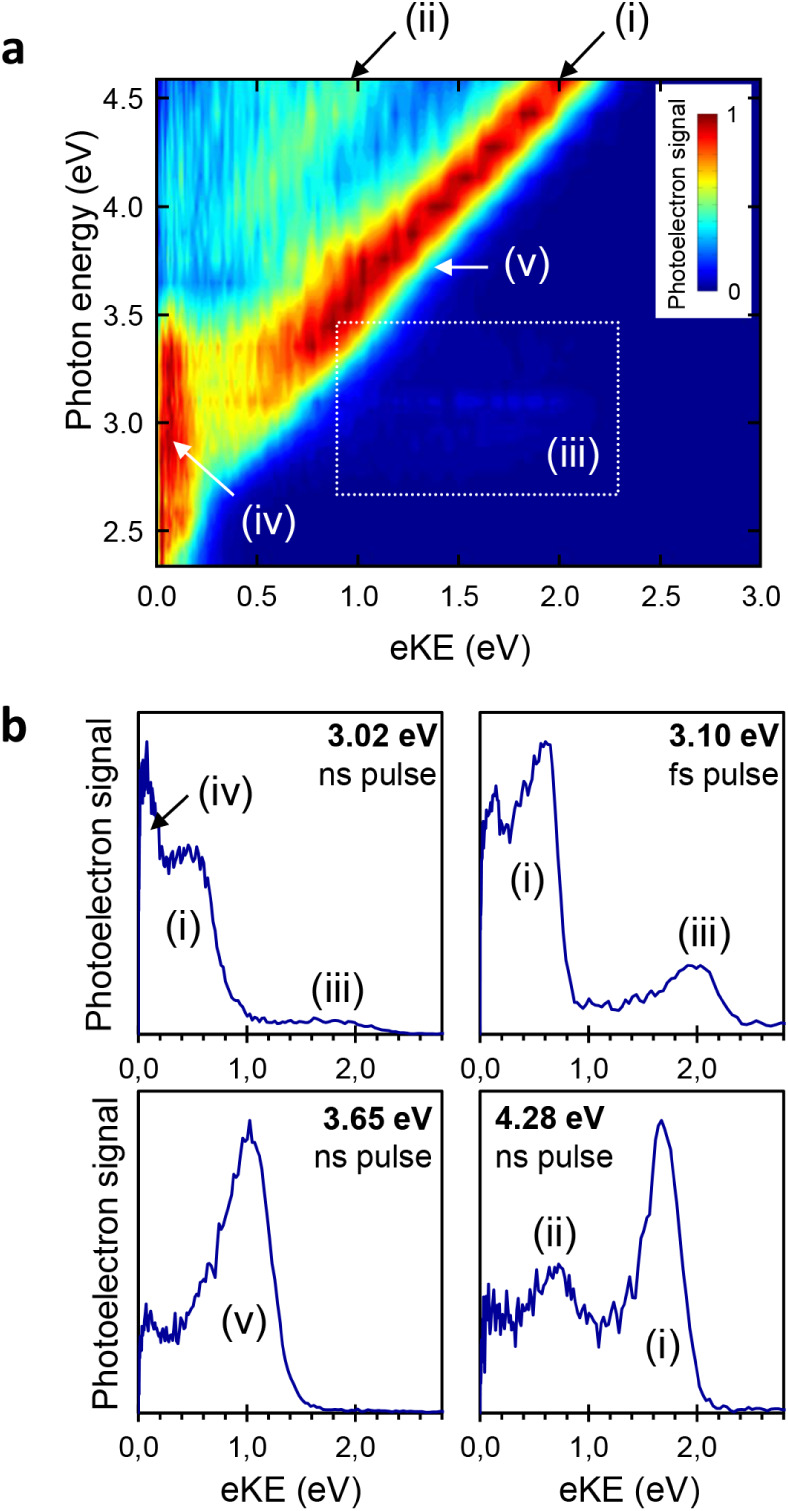
Photoelectron spectra of the meta-HBDI anion. (a) Two-dimensional photoelectron spectrum recorded with nanosecond laser pulses. (b) One-dimensional spectral cuts at specified excitation energies. For comparison, the spectrum at 3.10 eV was acquired with femtosecond pulses, contrasting with the 3.02 eV spectrum measured using ns pulses.

**Figure 4:**
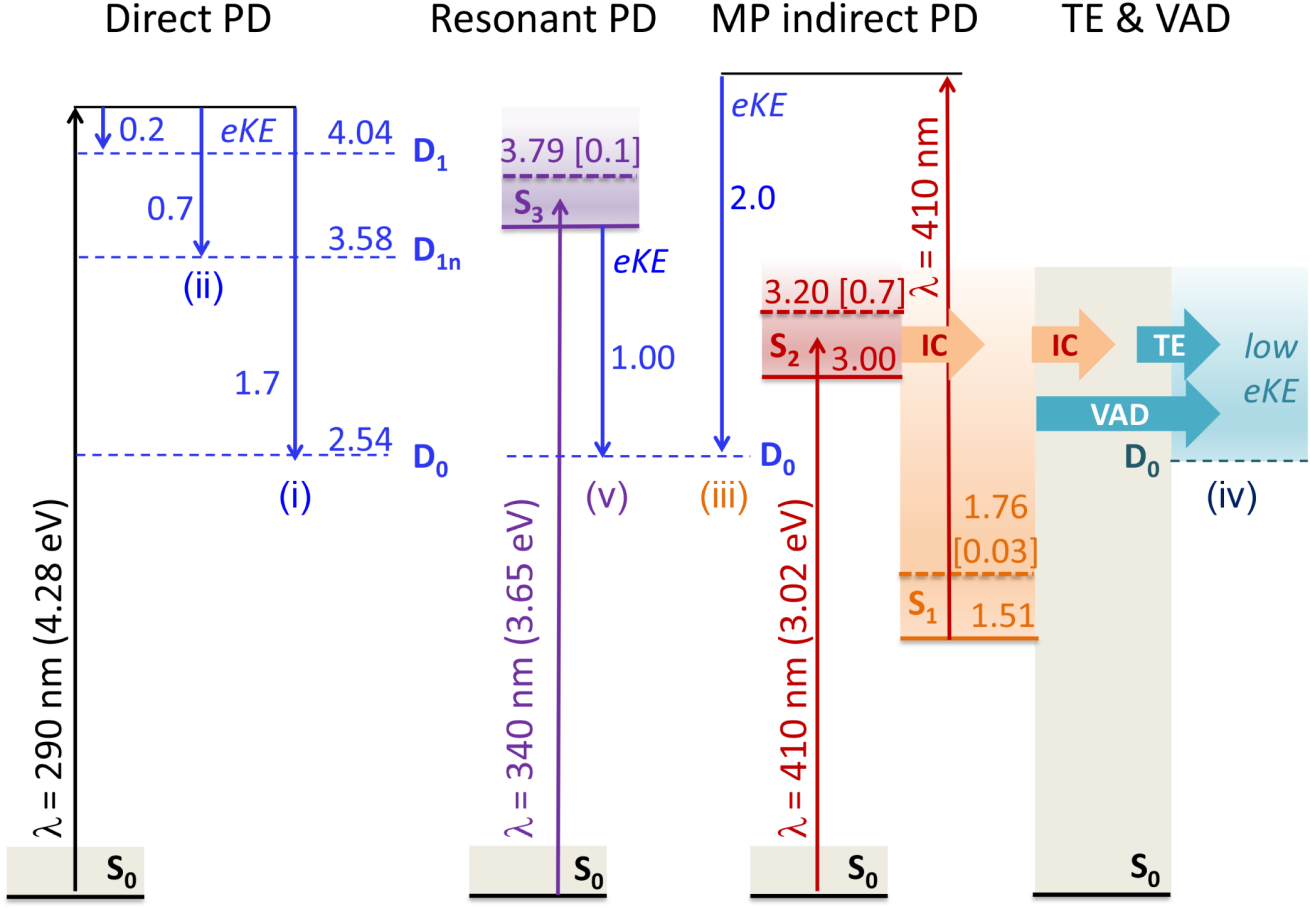
Wavelength-dependent photodetachment (PD) mechanisms for the meta-HBDI anion. The energy-level diagram, calculated at the XMCQDPT2/SA(3)-CASSCF­(16,14)/(aug)-cc-pVDZ level, shows pathways for different excitation energies: direct PD; resonant PD through the S_3_ shape resonance; multiphoton (MP) indirect PD via the S_1_ state after internal conversion (IC) from the S_2_ Feshbach resonance; thermionic emission (TE) from S_0_; and vibrational autodetachment (VAD) from S_1_. Channels (*i*) – (*v*) are identified in the experimental 2D photoelectron spectrum (Figure 3). Energies (in eV) and oscillator strengths (in brackets) are provided for key transitions. Adiabatic and vertical energies are shown as solid and dashed lines, respectively, with colored areas representing the vibrational manifold. Blue arrows indicate the electron-detachment process, labeled with the resulting electron kinetic energy (eKE).

We have shown that action-absorption spectroscopy has direct spectroscopic access to the near-IR dark S_1_ state of charge-transfer character. The presence of this electronically bound state is also confirmed by photoelectron spectroscopy. It is observed through a multiphoton indirect photodetachment process: initial excitation populates the bright S_2_ state above the detachment threshold, followed by internal conversion to the S_1_ state and subsequent electron detachment via absorption of a second photon. From the presence of the low-eKE part in the two-dimensional photoelectron spectrum, it also follows that internal conversion from S_2_ competes with electron autodetachment out of this resonance state. In the following, we present direct measurements of the S_2_ and S_1_ lifetimes using femtosecond pump–probe spectroscopy that integrates time-resolved action-absorption and photoelectron detection.

### Dynamics of Excited States

The femtosecond pump–probe technique combined with time-resolved action-absorption spectroscopy enables simultaneous monitoring of both excited-state decay and ground-state recovery. Figure 5 shows the measured prompt and delayed photoinduced yields of neutrals. The fast ∼100 fs decay of the prompt signal is attributed to ultrafast internal conversion from S_2_ to S_1_. The slower 94 ps decay, appearing as the long-lived decaying component of the prompt response, is attributed to the decay out of S_1_. It corresponds to the repopulation of the S_0_ ground state, fully consistent with the recovery dynamics revealed in the delayed action.

**Figure 5:**
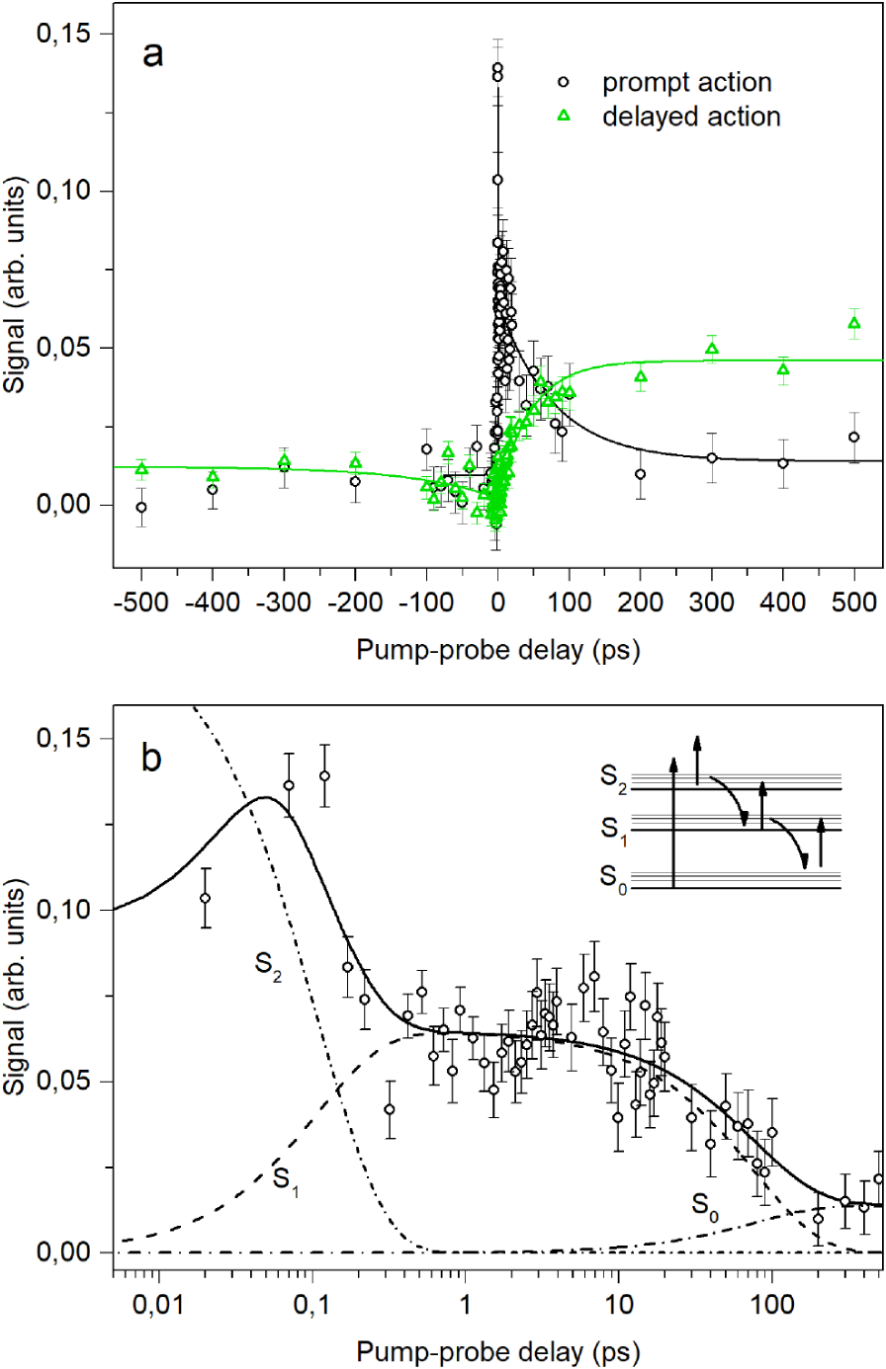
Excited-state lifetime measured in pump–probe experiments at the ion storage ring SAPHIRA. The molecules were first excited into S_2_ by a 400 nm pump pulse and then probed by a 800 nm probe pulse. The experimental setup enables the detection of prompt (within the first 10 μs after photoexcitation) as well as delayed (after 10 μs up to several ms after photoexcitation) photofragmentation. The upper graph (a) shows the prompt and delayed fragmentation as a function of the pump–probe delay on a linear time scale. While the lifetime of S_2_ and S_1_ can be probed by detecting prompt fragmentation, the delayed action shows the ground state recovery. In the lower graph (b), the signal of the prompt action is plotted on a logarithmic time scale and shows that the decay consists of a fast and a slow component. The fast decay of 100 fs ± 26 fs corresponds to the fast relaxation out of S_2_ with subsequent trapping in S_1_ for 94 ps ± 11 ps. The solid line represents a fit through the data points using a rate model indicated by the inserted schematic. The dashed lines show the calculated population of S_2_, S_1_ and S_0_.

The time-resolved photoelectron spectrum is plotted in Figure 6. It is evident that also here a very fast as well as a long-lived component exist. The fast component, which has a kinetic energy extending to ∼1.7 eV also decays within the first 100 fs. The spectrum of this feature is consistent with detachment from the S_2_ state. The S_2_-signal decay is commensurate with the appearance of a new spectral feature with a kinetic energy peaking around 0.45 eV, corresponding to a binding energy of about 1.1 eV. This can be assigned to the S_1_-excited state, which lies about 1.2 eV lower than the S_2_ state, according to Figure 1. As soon as the electronic ground state is reached, the absorption of a 1.55 eV (800 nm) probe photon does not suffice to lead to electron detachment. The S_2_ excited-state signal is observed to decay with a lifetime of ∼90 fs, which is in excellent agreement with the measurement by the action-absorption spectroscopy technique.

**Figure 6:**
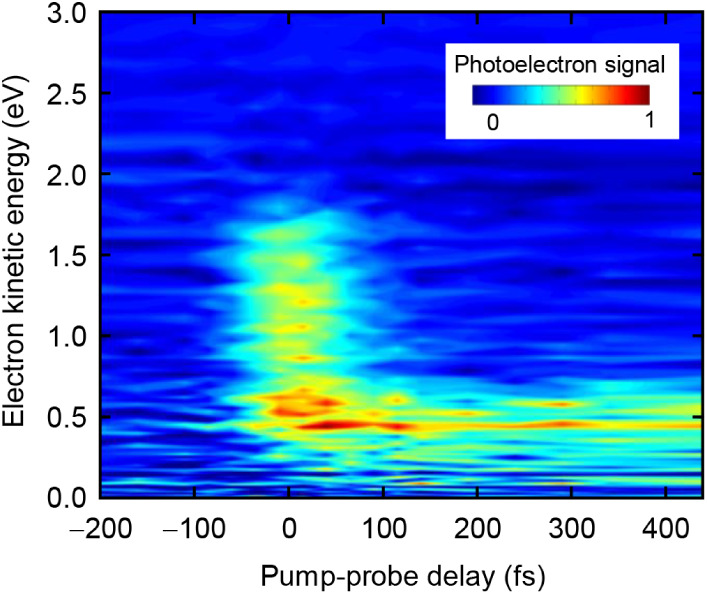
Photoelectron yield in pump–probe measurements (400 nm + 800 nm). The signal between 0.5 and 1.5 eV decays rapidly and is linked to the relaxation out of the S_2_ state. The signal at 0.4 eV remains for a longer time due to population trapping in the dark S_1_ state from the S_2_ state. The ground-state recovery is not detected due to insufficient photon energy in the 800 nm probe pulse.

### Trapping Mechanism

The experimental results are rationalized by the aid of *ab initio* calculations. We find that the S_1_ state of the meta-HBDI anion is optically dark in the Franck–Condon region, whereas the S_0_ → S_2_ transition is bright (see Figure 4). The calculated vertical excitation energy of the S_0_ → S_1_ transition is 1.76 eV (704 nm) with an oscillator strength of 0.03. The red-shifted value and the very low intensity of the first excitation are attributed to its nature connected to the transfer of charge and electron density from phenolate to the imidazolinone ring. The second transition is the brightest in the absorption spectrum, with a calculated vertical excitation energy of 3.2 eV (387 nm) and an oscillator strength of 0.7. It involves a local redistribution of electron density within the π-system of the imidazolinone ring. Remarkably, the bright S_0_ → S_2_ transition in meta-HBDI is blue-shifted relative to the bright transition in the para anion (vertical excitation energy of 2.52 eV with an oscillator strength of 1.1). This can be readily understood using a particle-in-a-box model: the effective conjugation length of the absorbing subsystem (Im=C) is shorter in the meta isomer, increasing the energy-level spacing and shifting the transition to higher energy. In contrast, the full conjugation of the para anion results in the red-shifted absorption. Conversely, the dark S_0_ → S_1_ charge-transfer transition in meta-HBDI is significantly red-shifted compared to the bright transition in the para anion. This red shift arises from enhanced interaction between the two subsystems upon excitation.

In the present study, ZITA action-absorption spectroscopy of the meta-HBDI anion complexed with betaine directly confirms the high charge-transfer character of the S_1_ state and the low charge-transfer character of the S_2_ state. The energetics of the S_1_ state is also confirmed experimentally. In action-absorption spectroscopy, a broad absorption profile between 550–700 nm is assigned to direct excitation of S_1_. The broad Franck–Condon envelope of this band is characteristic of a charge-transfer transition. Furthermore, photoelectron spectroscopy gives a vertical electron binding energy of 1.1 eV for the S_1_ state. When combined with the measured VDE­(D_0_) of 2.6 eV, this yields an S_0_ → S_1_ adiabatic excitation energy of 1.5 eV, a value in excellent agreement with the calculated result of 1.51 eV.

Due to effective decoupling of the two isoelectronic subsystems in meta-HBDI, the two bridge bonds are inequivalent in the ground state. The C–C and C=C bridge bond lengths are 1.44 and 1.37 Å, respectively, as calculated using the PBE0/(aug)-cc-pVDZ method. Analysis of the electron density in the S_1_ and S_2_ states reveals that excitation reduces the π-bond order of the C=C bond adjacent to the imidazolinone ring by a factor of 1.5 in S_1_ and 3.2 in S_2_ (see Figure S4). The substantial bond-order reduction in the S_2_ state points to a relaxation pathway involving rotation about this bond, leading to a S_2_/S_1_ conical intersection (CI). Despite the marked differences in electron density distribution between S_2_ and S_1_, their dominant valence resonance structures are coupled (Figure S5). This excited-state resonance mixes the two isoelectronic π-subsystems, equalizing the probability of negative charge (or radical) localization on the phenolate oxygen and the bridge carbon, and thus effectively redefines the partitioning of the π-system. Rotation about the bridge C=C bond emerges as the key reaction coordinate that drives the system toward the S_2_/S_1_ CI by balancing the charge localization probabilities between the two states (Figure S6). Consequently, ultrafast excited-state dynamics is expected to originate from the bright S_2_ state, implicating a highly efficient internal conversion channel via this conical intersection.

Three conical intersections are located that interconnect the S_2_, S_1_, and S_0_ states, which are relevant to the relaxation dynamics of the meta-chromophore in the gas phase. Figure 7 shows the potential energy surfaces of S_2_, S_1_, and S_0_ in the branching plane of the S_2_/S_1_ CI. The initial relaxation through the peaked S_2_/S_1_ CI occurs barrierlessly (Figure S7) and is therefore very fast, in agreement with the experimental finding. The decay results in population of the S_1_ state despite the open electron-detachment channel. However, a bifurcation at the S_2_/S_1_ CI funnels the S_1_ population toward two distinct conical intersections with the ground state (Figure 7). These two CIs are differentiated by the character of the first excited state: one acquires the charge-transfer character of the dark S_1_ (CT) state, while the other retains the character of the bright, locally excited S_2_ (Loc) state. Therefore, they are labeled as S_1_/S_0_ (CT/S_0_) and S_2_/S_0_ (Loc/S_0_), respectively, based on the character of each state at the ground-state equilibrium geometry.

**Figure 7:**
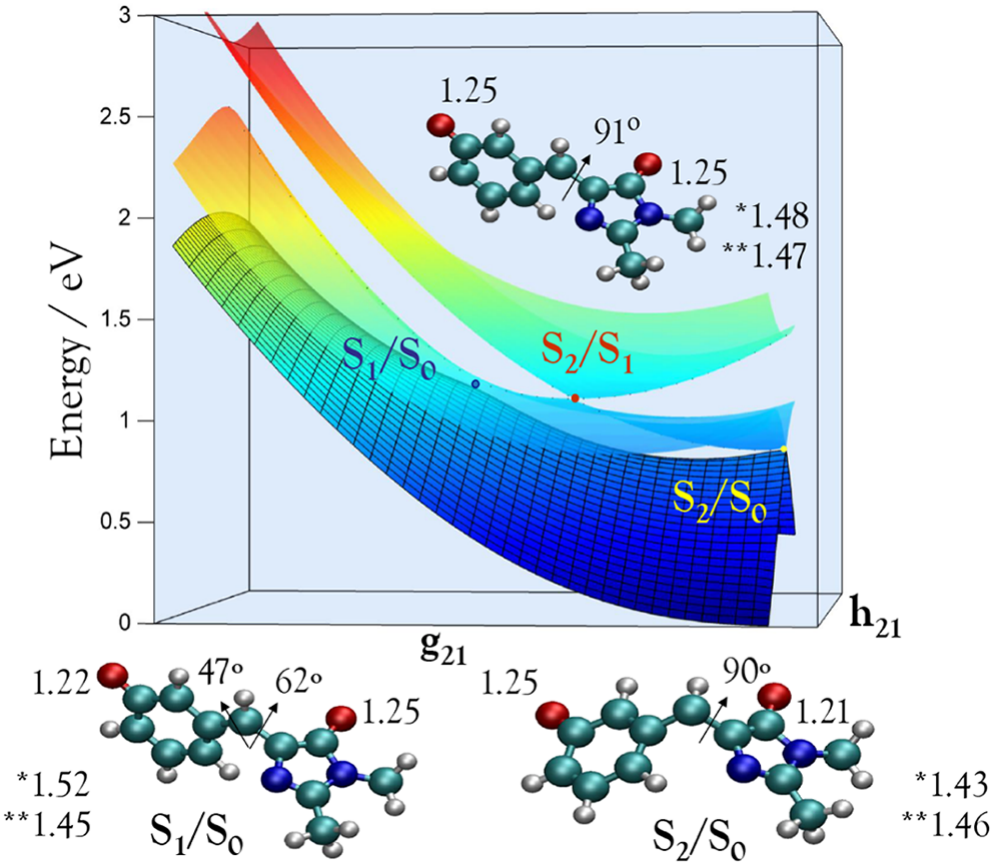
CASSCF­(16,14)/(aug)-cc-pVDZ potential-energy surface illustrating the conical intersections governing the excited-state dynamics of the meta-HBDI anion. The surface is plotted in the branching plane of the S_2_/S_1_ conical intersection, spanned by the gradient difference vector (**g**_21_) and the nonadiabatic coupling vector (**h**_21_). Also shown are the MECI structures, with key torsional angles in the bridge moiety, O–C bond lengths, and bridge C–C (*) and C=C (**) bond lengths (in Å) indicated (see also Figure S8). This representation highlights how nuclear motion along these directions defines the nonradiative decay along two competing branches involving three conical intersections. Note that the intermediate S_1_ state of the CT character traps the excited-state population at the planar equilibrium geometry. The S_1_/S_0_ (CT/S_0_) MECI on the dark branch is the lowest-energy pathway for internal conversion to the ground state, while the S_2_/S_0_ (Loc/S_0_) MECI lies 0.14 eV higher in energy (see Figure 8).

The calculated energy-level diagram identifies the location of the minimum-energy conical intersections (MECIs) governing the nonadiabatic transitions between the S_2_, S_1_, and S_0_ states (see Figure 8). Following efficient internal conversion from S_2_, the meta-HBDI anion becomes trapped in the planar equilibrium geometry of the S_1_ state. This trapping occurs because the S_2_/S_0_ (Loc/S_0_) and S_1_/S_0_ (CT/S_0_) MECIs lie 0.55 and 0.41 eV higher in energy, respectively. The two decay pathways are structurally distinct: the S_2_/S_0_ (Loc/S_0_) CI involves rotation about the double C=C bridge bond, a pathway that could lead to isomerization. In contrast, the S_1_/S_0_ (CT/S_0_) CI is characterized by twisting about both bridge bonds and significant pyramidalization of the bridge carbon (see Figures 7 and S8). In the gas phase, the relaxation proceeds predominantly along this latter pathway, resulting in a long-lived nonfluorescent trapping state that ultimately decays to the ground state without isomerization.

**Figure 8:**
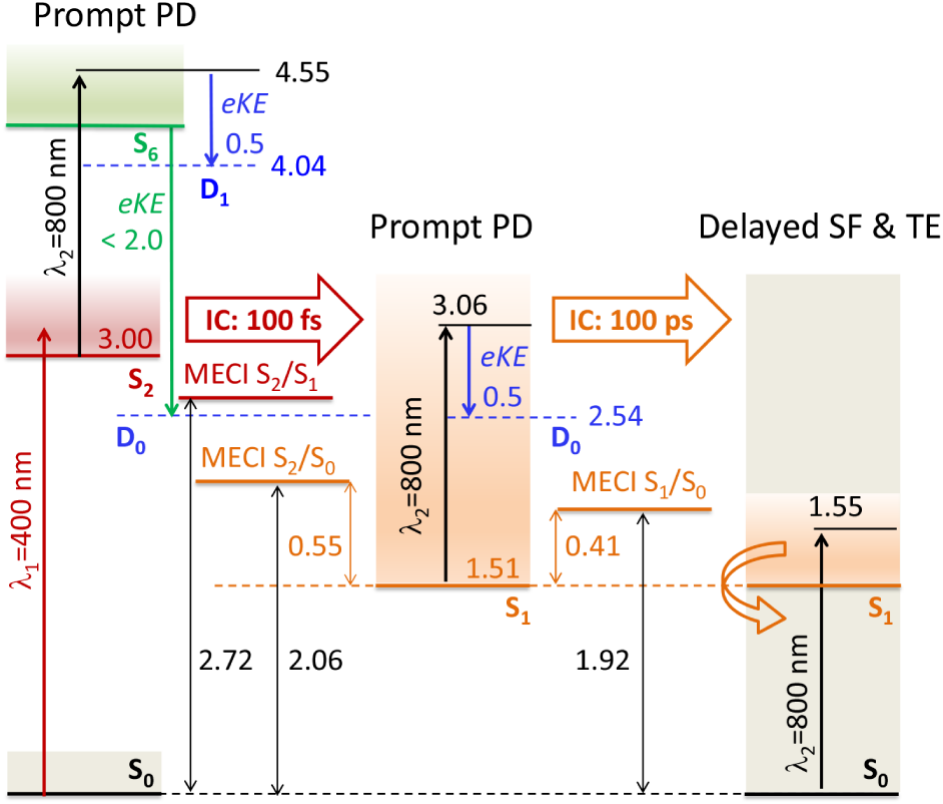
Trapping mechanism in the meta-HBDI anion. The XMCQDPT2/SA(3)-CASSCF­(16,14)/(aug)-cc-pVDZ diagram shows relative energies of key electronic states and minimum-energy conical intersections (MECI) between the S_2_, S_1_, and S_0_ states. Shown are also the prompt and delayed channels in pump (λ_1_)–probe (λ_2_) spectroscopy. The excited-state decay is registered both by measuring the energy of emitted electrons and by counting neutrals in prompt photodetachment (PD) channels. The ground-state recovery is measured as a delayed signal through counting neutrals following statistical fragmentation (SF) and thermionic emission (TE) from S_0_. Energies are in eV.

It is instructive to compare the two types of CIs in meta-HBDI along the dark (CT/S_0_) and bright (Loc/S_0_) branches with those previously characterized in para-HBDI.^20,39^ In both isomers, population of the relevant excited state substantially reduces the order of the bridge bond adjacent to the imidazolinone ring, enabling rotation-based internal conversion. A key distinction remains: in meta-HBDI, the two bridge bonds are inequivalent in the ground state and become nearly equalized only upon excitation, whereas in para-HBDI, resonance delocalization ensures their equality already in the ground state. In para-HBDI, the two S_0_/S_1_ conical intersections arise from an asymmetric allyl anion model of the bridge moiety, where distinct electron affinities of the phenolate and imidazolinone rings define the asymmetry. The two twisting pathways are nonequivalent: rotation about the bond adjacent to the imidazolinone ring brings the surfaces into close proximity, while rotation about the bond adjacent to the phenolate ring requires additional twisting and pyramidalization at the central carbon, yielding a higher-energy hula-twist geometry. In meta-HBDI, the two types of conical intersections originate from branching within the first excited state and its distinct electronic characters. The CT state, characterized by a substantial charge accumulation on the bridge carbon, formally corresponds to the hula-twist type. In contrast, the locally excited state, featuring a negative charge localization on the phenolate moiety and a pronounced reduction in the π-bond order of the C=C bridge bond, leads to a conical intersection of the first type. Owing to the distinct electronic structures of the two isomers, the relative energies of these conical intersections also differ.

The photoinduced dynamics, studied using femtosecond pump–probe spectroscopy, is characterized by two complementary methods: (1) time-resolved action-absorption spectroscopy, which monitors prompt and delayed neutrals; and (2) photoelectron spectroscopy, which detects prompt electrons generated by direct photodetachment from the excited states. Direct detachment out of S_2_ should comply with the Slater rules. In other words, two-electron processes are forbidden in the dipole approximation. Therefore, following 400 nm excitation of S_2_ in meta-HBDI, an 800 nm probe photon can detach an electron from S_2_ with the most probable eKE of 0.5 eV (D_1_), leaving the neutral with 0.1 eV of vibrational energy (Figure 8). Alternatively, the probe photon can promote the anion from S_2_ to a higher-lying resonance at ∼4.6 eV, which autodecays, producing a broad electron kinetic energy distribution up to 2.0 eV across all open continua (D_1_, D_1*n*_, and D_0_). The experimental eKE distribution of up to ∼1.7 eV observed at early times (Figure 6) is consistent with both direct and resonant photodetachment pathways, which leave some vibrational energy in the neutral core. This is supported by the XMCQDPT2 calculations, which identify an excited state at 4.6 eV at the ground-state equilibrium geometry, with a non-negligible oscillator strength of 0.06 for the S_2_ → S_6_ transition (see Figure 8).

If internal conversion to S_1_ occurs first, the same probe detaches an electron from S_1_, producing electrons with an energy of 0.5 eV (D_0_) and a vibrationally hot neutral with 1.59 eV of excess energy. The significant narrowing of the eKE distribution peaking at 0.45 eV after 100 fs (Figure 6) confirms the direct photodetachment pathway. Repopulation of the S_2_ state from S_1_ by the 800 nm probe is negligible due to the vanishing oscillator strength of this transition (Figure 8). Following internal conversion to S_0_, the 800 nm excitation leads to repopulation of the electronically bound S_1_ state, resulting in slow statistical fragmentation (SF) and (thermionic emission) TE from the hot ground state after internal conversion. The excited-state decay and ground-state recovery align well with the prompt and delayed signals observed in our pump–probe action-absorption experiments (Figure 5).

The excited-state population is trapped in S_1_ due to the presence of a barrier along the minimum-energy pathway that leads from the planar S_1_ equilibrium structure to the highly twisted S_1_/S_0_ (CT/S_0_) conical intersection. The XMCQDPT2/SA(3)-CASSCF­(16,14)/(aug)-cc-pVDZ barrier of 0.41 eV is associated with reaching the lowest-lying S_1_/S_0_ CI with a sloped topography along the dark branch (see Figures 8 and S8). Quasi-equilibrium theory for a microcanonical ensemble predicts a statistical lifetime of 98 ps for the S_1_ excited state at 300 K after 400 nm excitation (Figures S9–S10), a value that agrees perfectly with our experimental findings.

## Conclusions

The spectroscopy and excited-state dynamics of meta-HBDI were studied by performing action spectroscopy and femtosecond pump–probe experiments. Our action-photoabsorption spectroscopy studies in vacuum directly revealed the presence of an optically dark S_1_ state, originating from the π-π* transition with a CT character. We assessed the strong CT character of the S_0_ → S_1_ transition by measuring the blue-shift of the S_1_ absorption band of meta-HBDI complexed with the betaine zwitterion. Femtosecond pump–probe experiments revealed ∼100 fs ultrafast relaxation out of the excited S_2_ state, followed by population and trapping in the S_1_ state for ∼100 ps. Our experimental observations were rationalized by high-level quantum-chemistry calculations. Three conical intersections were found that interconnect the S_2_, S_1_, and S_0_ states, which are relevant to the relaxation dynamics of the meta-chromophore in the gas phase. Although dark, the S_1_ state can be populated from the higher-lying bright S_2_ state by internal conversion that involves conical intersections. Importantly, this extremely efficient process outcompetes and thus suppresses electron detachment from the S_2_ electronic resonance. The actual time scale for populating the dark state depends on the potential-energy topography, and the trapping lifetime is determined by energy barriers, along the minimum-energy pathway that leads from the planar S_1_-minimum structure to the highly twisted S_1_/S_0_ conical intersection. The charge-transfer character of the low-lying dark state provides a direct handle to control its lifetime via the polar environment. This offers a clear design principle for engineering synthetic chromophores with tailored photophysical properties.

Our gas-phase action-absorption and photoelectron spectroscopy study combined with high-level *ab initio* calculations has unequivocally identified a long-lived dark state that acts as an efficient trapping site in the meta-HBDI anion. This discovery and the detailed insight into the charge-transfer character establish a mechanistic framework for understanding photostability, in which dark states act as a general “energy sink”. By dissipating excess electronic energy, these states suppress deleterious electron transfer and photodegradation following UV excitation. This principle, now revealed in the unperturbed chromophore, may extend to other environments, where similar states could be tuned to enhance photostability or, conversely, to design optical switches. Furthermore, it draws a compelling parallel to the function of dark states in carotenoids, which are critical for photoprotection. By providing direct, gas-phase spectroscopic access to these elusive states, our work establishes a new benchmark for unraveling complex excited-state landscapes, offering a fresh perspective on a cornerstone of biological photochemistry.

## Methods

This study employs a unique combination of gas-phase photoabsorption detection, femtosecond time-resolved action spectroscopy, and photoelectron spectroscopy, supported by high-level quantum chemical calculations to interpret the results.

### Action-Absorption Spectroscopy

Action-photoabsorption spectroscopy measurements of meta-HBDI were performed at room temperature at the ion-storage ring ELISA.^13,14^ Methanol-dissolved chromophores were brought into the gas-phase by electrospray ionization, injected into the storage ring and photoexcited by a pulsed nanosecond-laser system (EKSPLA, NT232–50-SH-SFG). The number of neutral fragments after photoexcitation was used as a measure of the absorption cross-section after normalization to the number of photons and stored ions.

### Pump–Probe Time-Resolved Action-Absorption Spectroscopy

The relaxation dynamics was investigated by exciting the molecules by fs-laser pulses in the SAPHIRA ion-storage ring at Aarhus University.^15,16,40^ Here the molecules were excited with a 400 nm fs pump-laser pulse into S_2_, and afterward probed by a 800 nm fs-laser pulse at a variable pump–probe delay. In addition to the short time delay between the two fs-laser pulses, the molecular-response time by dissociation was recorded by the ion-storage technique. Thus, as pump–probe signal (as a function of the probe–pulse delay) we used the time-resolved yield of neutral photofragments in the storage ring. The advantage of the ion storage ring technique is that it enables the registration of prompt action (fragmentation within the first 10 μs after photoexcitation) as well as delayed action. The counts of prompt neutral fragments after pump and (delayed) probe pulses were created largely by photodetachment rendering a neutral detectable product. The delayed action, on the other hand, appeared when the S_2_ state returned to the S_0_ ground state by internal conversion, where slow statistical fragmentation and thermionic electron emission occur, typically on the millisecond time scale.

### Frequency- and Time-Resolved Photoelectron Spectroscopy

Photoelectron spectroscopy measurements were carried out at Durham University.^17,18^ Briefly, electrons were detected and energy analyzed as a function of the photon energy to yield photoelectron spectra.^41^ In addition, the number of energy-selected photoelectrons were recorded as a function of pump–probe time delay to follow the excited-state relaxation dynamics.

### Ab Initio Calculations

High-level *ab initio* calculations were performed using the multistate multireference perturbation theory XMCQDPT2 ^34^ and complete active space self-consistent field method. The Firefly package^42^ was used for all quantum chemistry calculations. The S_1_ excited-state lifetime was computed as functions of ground-state temperature and excitation wavelength within the bright S_0_ → S_2_ transition using quasi-equilibrium theory for a microcanonical ensemble. The details are described in the Supporting Information.

## Supplementary Material









## Data Availability

The experimental data generated (raw photoelectron spectra) have been deposited and can be accessed at 10.5281/zenodo.19145478.

## Notes

Additional computational details, intramolecular rotation potential in S_0_, character of electronically excited states, intramolecular rotation potential in S_2_, XMCQDPT2 structures of conical intersections along the dark branch, calculated S_1_ excited-state lifetimes (PDF)
Animation of the gradient difference vector for the S1/S0 MECI (GIF)
Animation of the gradient difference vector for the S2/S0 MECI (GIF)
Animation of the gradient difference vector for the S2/S1 MECI (GIF)
