# Supplementary material for: Dark-State-Mediated Efficient Energy Trapping in a Model GFP Chromophore
Source: J Am Chem Soc. 2026 Apr 17;148(16):16750–9. doi: 10.1021/jacs.5c22023 (PMC13133902; doi:10.1021/jacs.5c22023)
Supplement: Supplementary file 1 [file ja5c22023_si_001.pdf]

Supporting Information: Dark-State-Mediated Efficient Energy Trapping in a Model GFP Chromophore

Elisabeth Gruber,[†] Lars H. Andersen,[*,†] Laurence H. Stanley,[‡] Jan R. R. Verlet,[*,‡] Ivan S. Avdonin,[¶] and Anastasia V. Bochenkova[*,¶]

†*Department of Physics and Astronomy, Aarhus University, 8000 Aarhus C, Denmark.*

‡*Department of Chemistry, Durham University, Durham DH1 3LE, United Kingdom*

¶*Department of Chemistry, Lomonosov Moscow State University, 119991 Moscow, Russia*

E-mail: LHA@phys.au.dk; j.r.r.verlet@durham.ac.uk; bochenkova@phys.chem.msu.ru

Contents

Computational details **S-3**

Ground-state intramolecular rotation **S-5**

Character of electronically excited states **S-6**

Intramolecular rotation in S_2 **S-12**

Conical intersections along the dark branch **S-13**

Calculated S_1 excited-state lifetimes **S-14**

References **S-15**

Computational details

The energy diagram of the excited and electron-detached (neutral) levels was constructed based on the calculated adiabatic and vertical transition energies using multiconfigurational quasi-degenerate perturbation theory XMCQDPT2. Ground-state geometry parameters were obtained using the PBE0/(aug)-cc-pVDZ method. The low-lying $\pi - \pi^*$ vertical excitation energies (VEE) of the anion were calculated at the XMCQDPT2/SA(7)-CASSCF(16,14)/(aug)-cc-pVDZ level of theory within the active spaces, which comprised all valence orbitals of π-type. The vertical detachment energy (VDE) of the meta-chromophore anion was calculated at the XMCQDPT2/SA(2)-CASSCF(14,14) level of theory within the (p-type d-aug)-cc-pVTZ basis set. The oxygen atoms were treated using the entire aug-spdf diffuse shells. The basis set was also augmented with an additional diffuse function of p-type with a particularly small exponent (10^{-10}) centered at the middle carbon atom. The π^* orbital of this highly diffuse shell was included in the active space and was used to mimic an electron-detachment process. The ground and ionized states were included in a state-averaging procedure. The XMCQDPT2 effective Hamiltonians were constructed in the frame of the reference spaces spanned by 9 zeroth-order wavefunctions.

The positions of higher-lying vertical detachment thresholds π^{-1} (D_n, n=1,2,..) for all anions were calculated relative to D_0 as vertical excitation energies in the corresponding radical species, using the XMCQDPT2/SA(6)-CASSCF(15,14)/(aug)-cc-pVDZ method. The positions of the first excited π^{-1} (D_1) as well as n^{-1} (D_{1n}) VDEs were also calculated in the frame of a slightly modified approach, XMCQDPT2/SA(2)-CASSCF(15,14)/(aug)-cc-pVDZ, where a state-averaging procedure included the ground state and a target-excited state only, within the corresponding active spaces. The variations in the state-averaging procedure within the π, π^* active space had only a small effect (less than 0.06 eV) on the VEEs to the first excited state D_1 of the radical. For the $n \rightarrow \pi$, π^* radical calculations, the active spaces were modified to include the n orbital of the phenoxide oxygen, whereas one occupied π-orbital with the largest occupation number was kept as doubly occupied

during the CASSCF procedure. The excluded orbital referred to a lone pair of electrons predominantly localized at the nitrogen atom of the amino group of the heterocyclic ring.

The S_1 excited-state equilibrium geometry parameters, minimum-energy conical intersections, and topographies around the CIs were obtained at the SA(3)-CASSCF(16,14)/(aug)-cc-pVDZ level of theory and then refined using the multiconfigurational quasi-degenerate perturbation theory XMCQDPT2[3]/SA(3)-CASSCF(14,13)/(aug)-cc-pVDZ. The reorganization energy in the S_2 state was estimated based on the XMCQDPT2/SS-CASSCF(14,13)/(aug)-cc-pVDZ excited-state gradient calculations, where the π-active space was reduced by eliminating one occupied orbital with the largest occupation number.

The S_1 excited-state lifetime was computed as functions of ground-state temperature and excitation wavelength within the bright $S_0 \to S_2$ transition using quasi-equilibrium theory (QET) for a microcanonical ensemble. Within the QET framework, the microcanonical rate constant for a total energy E is defined as

$$k(E) = \frac{W^{\ddagger}(E - E_0^{\ddagger})}{h\rho(E)} \tag{1}$$

where $W^{\ddagger}(E - E_0^{\ddagger})$ is the sum of vibrational states above the barrier $E_0^{\ddagger}$ at the transition state; $\rho(E)$ is the density of vibrational states for the reactant; and h is the Planck constant. The sum and density of states were calculated using the Beyer-Swinehart (BS) algorithm. The excited-state lifetime was estimated as the inverse of the microcanonical rate constant. The key parameter was the potential energy barrier, derived from XMCQDPT2//SA(3)-CASSCF(16,14)/(aug)-cc-pVDZ energies of the planar S_1 minimum and the lowest-energy S_1/S_0 conical intersection. All geometries and harmonic frequencies at the S_1 minimum were obtained at the SA(3)-CASSCF(16,14)/(aug)-cc-pVDZ level of theory.

Ground-state intramolecular rotation

Figure S1 shows the calculated potential that hinders rotation about the single C-C bridge bond in meta-HBDI in the ground electronic state. Two rotamers are present, separated by 0.05 eV with an interconversion barrier of 0.46 eV. The calculated vertical detachment energy (VDE) is identical for both rotamers (2.5 eV) at their equilibrium geometries. However, rotation along the interconversion coordinate lowers this energy by 0.2 eV in the transition state. Given the relatively high interconversion barrier and the nearly identical absorption and detachment energies of the two rotamers in their planar forms, we have used the lowest-energy rotamer A exclusively throughout this study.

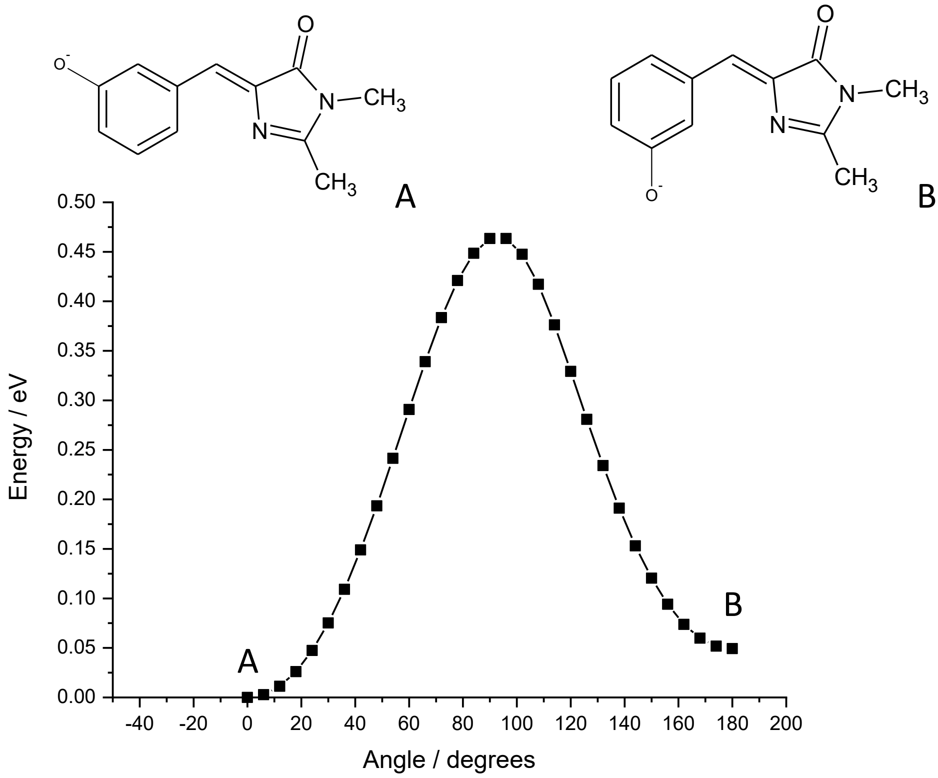

Figure S1: MP2/(aug)-cc-pVTZ potential that hinders rotation about the single C-C bridge bond in meta-HBDI in the ground electronic state. Note that two rotamers are present, with an interconversion barrier of 0.46 eV and an energy difference of 0.05 eV between them.

Character of electronically excited states

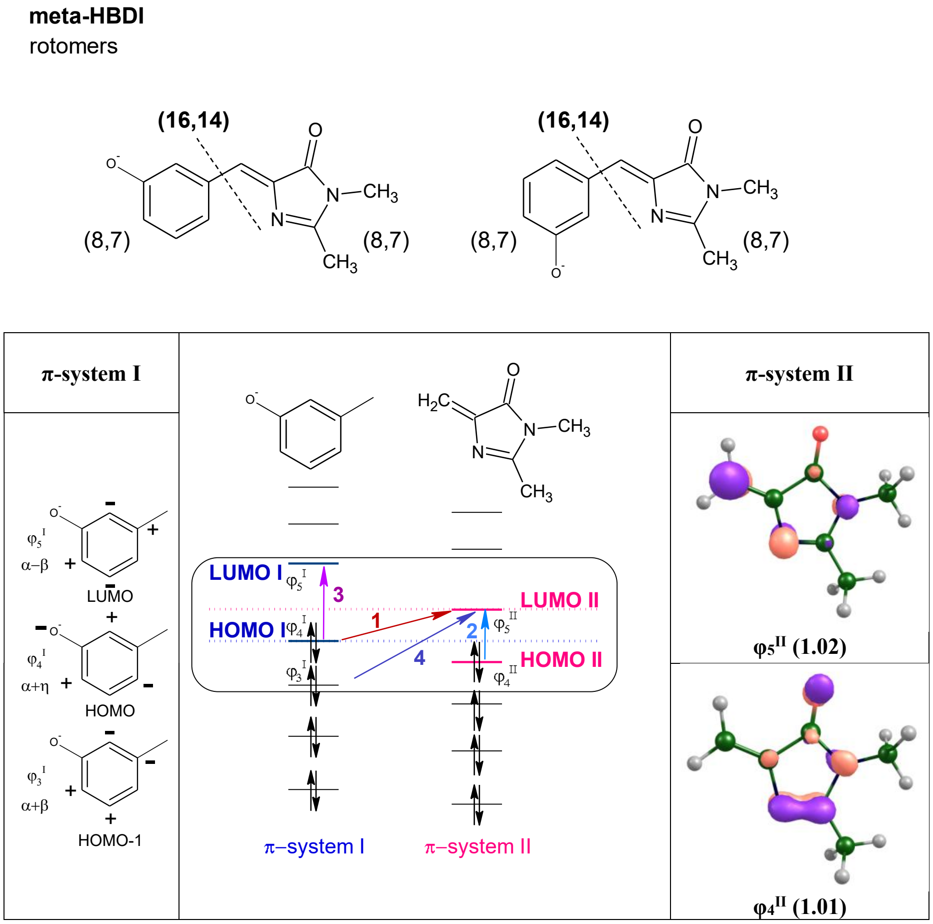

Figure S2: Schematic of electronic transitions in the meta-HBDI anion, illustrating the partition of its π-system into two isoelectronic parts. The central panel depicts intrasystem (2, 3) and intersystem (1, 4) excitations. The left panel shows the Hückel model for π-system I, while the right panel displays the XMCQDPT2 natural orbitals primarily involved in the lowest-energy bright transition in π-system II, with the occupation numbers given in brackets.

The meta-HBDI anion has 16 π-electrons distributed across 14 orbitals. Its electronic structure is best described by considering two isoelectronic (8,7) subsystems (see Fig. S2). The first (π-system I) comprises the negatively charged phenolate-ring moiety, while the second (π-system II) consists of the neutral imidazolinone ring and the bridge carbon atom.

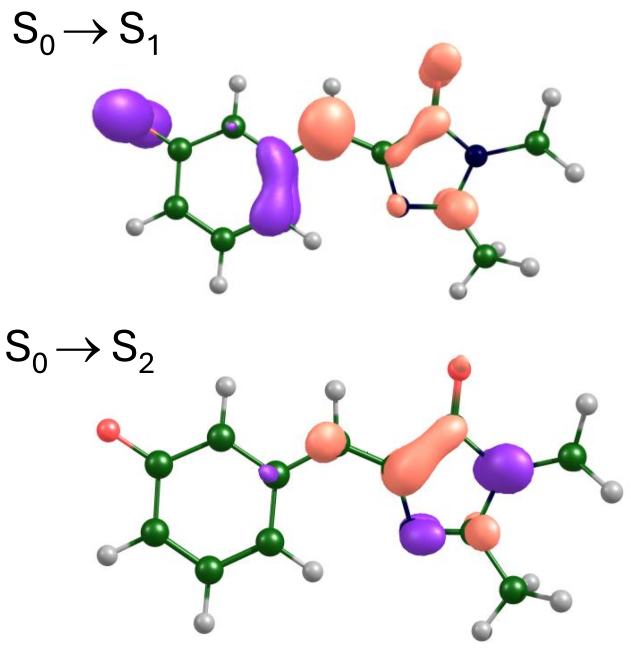

Figure S3: Zero-order XMCQDPT2 electron density redistribution upon the $S_0 \rightarrow S_1$ and $S_0 \rightarrow S_2$ transitions. An increase in electron density in the excited state is shown in pink, and a decrease is shown in purple.

The electronic transitions of the full system can thus be characterized as intra- and inter-subsystem excitations. The highest occupied molecular orbital (HOMO) of the entire system belongs to π-system I, while the lowest unoccupied molecular orbital (LUMO) is localized in π-system II. This spatial separation of frontier orbitals underpins excited-state properties of meta-HBDI.

The meta-anion absorption spectrum is characterized by a distinct $\pi\pi^*$ transition of the charge-transfer character (CT). The red-shifted value and the very low intensity of the first excitation (λ_{max}=704 nm with an oscillator strength of 0.03) are attributed to its nature connected with the transfer of charge and electron density from the Ph-ring to the Im-ring moiety upon the $S_0 \rightarrow S_1$ transition (see Fig. S3). The dark S_1 state originates from a one-electron excitation between the two isoelectronic π-systems. These systems are effectively decoupled due to the absence of valence resonance structures in the ground electronic state

	Ground state	Dark charge-transfer state	Bright state
pHBDI anion	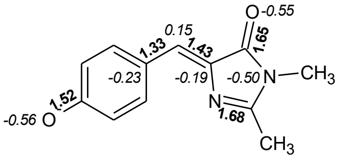<br>charge I: -0.64; II: -0.36	–	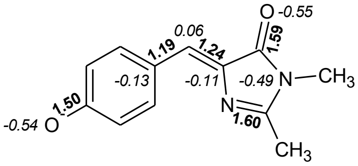<br>charge I: -0.53; II: -0.47
mHBDI -anion	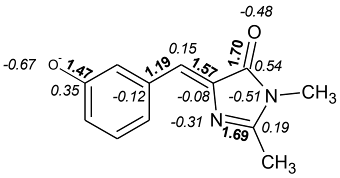<br>charge I: -0.89; II: -0.11	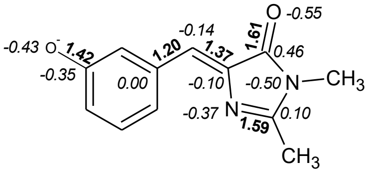<br>charge I: -0.29; II: -0.71	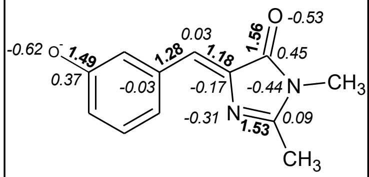<br>charge I: -0.53; II: -0.47

Figure S4: meta-HBDI and para-HBDI excited-state properties at the ground-state equilibrium geometry based on the zero-order XMCQDPT2 density: bond orders (in bold), Mulliken partial charges (in italic), and total Mulliken charges of subsystems I and II.

of the meta-HBDI anion. The second transition, the brightest in the absorption spectrum (λ_{max}=387 nm with an oscillator strength of 0.7), involves the local electron density redistribution within π-system II (see Fig. S3). It populates the same unoccupied orbital as the first excited state. In contrast, the next intra-system transition is localized in π-system I, involving excitation confined to the phenolate ring (λ_{max}=327 nm with an oscillator strength of 0.06).

When LUMO of π-system II is populated, the bridge carbon atom gains additional negative charge, and the π-bond order of the C=C bond adjacent to the imidazolinone ring is

dramatically reduced – by a factor of 1.5 in the S_1 state and 3.2 in the S_2 state (see Fig. S4). This equalizes the two bridge bonds, a process underscored by a concomitant slight increase in the order of the second bridge bond. These structural changes provide initial insights into the relaxation pathways, involving rotation around the bridge bonds. Specifically, the significant bond-order reduction in the S_2 state indicates a high probability of efficient internal conversion via rotation around this C=C bond.

The electronic structure of the para-HBDI anion differs from that of its meta counterpart. In the para isomer, two resonance structures delocalize the negative charge over both oxygen atoms, equalizing the lengths and bond orders of the two bridge bonds in both the ground and first excited states. Upon the the $S_0 \rightarrow S_1$ excitation, XMCQDPT2 calculations show that the π-bond orders of both bridge bond decrease by a factor of approximately 1.8, from 0.43 to 0.24 for the bond adjacent to the imidazolinone ring, and from 0.33 to 0.19 for the bond adjacent to the phenol ring (see Fig. S4). This excitation-induced change in electron density can be understood through a simple allyl-anion Hückel model that mimics the bridge moiety. The model not only reproduces the electron density redistribution quantitatively but also predicts a factor-of-two decrease in the order of the totally symmetric π-bonds (from 0.7 to 0.35).[S1] In this picture, the central carbon atom acquires additional negative charge from its neighbors – a redistribution reflected in the molecular orbitals involved in the transition, which correlate with the frontier orbitals of the Hückel allyl system. The allylic model thus provides an intuitive framework for understanding key photophysical properties of the para-HBDI anion. It accounts for the bright character of the S_1 state and explains the efficiency of non-radiative relaxation pathways, which proceed through S_1/S_0 conical intersections accessed by rotation about the bridge bonds in the excited state.

The analysis of the electronic structure of the meta-HBDI and para-HBDI anions also reveals a similarity: in both isomers, population of the relevant excited state leads to negative charge accumulation on the bridge carbon atom and a pronounced reduction in the order of the bridge bond adjacent to the imidazolinone ring, facilitating rotation-based internal

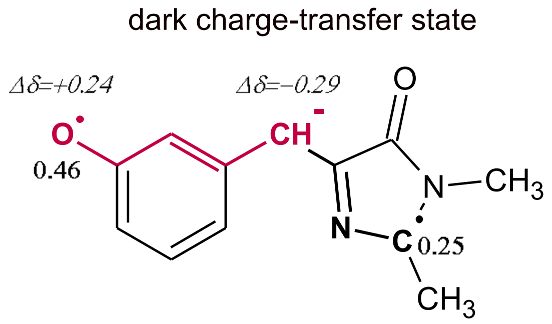

Figure S5: Dominant resonance structures of the meta-HBDI anion at the ground-state equilibrium geometry, illustrating the electronic configurations of the S_1 (dark) and S_2 (bright) excited states. Free valences arising from the excitation are indicated in bold, while changes in Mulliken partial charges relative to the ground state are shown in italic. The valence anaylsis is based on the zero-order XMCQDPT2 electron density. The reference partial charges are shown in Fig. S4.

conversion. However, an important distinction remains: in meta-HBDI, the two bridge bonds are inequivalent in the ground state and become equalized only upon excitation, whereas in para-HBDI, resonance delocalization ensures their equality already in the ground state.

In the meta-HBDI anion, the analysis of electron density and charge distribution in the S_2 and S_1 states provides a rationale for the S_2/S_1 conical intersection pathway, involving rotation around the bridge C=C bond. Despite the pronounced difference in the electron density distribution in S_2 and S_1, their dominant valence resonance structures are coupled (see Fig. S5). This excited-state resonance scrambles both π-systems I and II, equalizing the probability of negative charge (or radical) localization on the phenolate oxygen and the bridge carbon. Consequently, the resonance changes the effective partition of the π-system, by extending subsystem I and shortening subsystem II by one carbon atom. While the contribution of each resonance structure to the other state is negligible at the ground-state geometry, our analysis indicates that rotation around the C=C bond adjacent to the imida-

zolinone ring is the key coordinate for equalizing their contributions. This rotation disrupts conjugation in subsystem II, causing the energies of the frontier orbitals of the two subsystems to converge monotonically – the HOMO of subsystem II rises while that of subsystem I falls (Fig. S6). The degeneracy point corresponds to the S_2/S_1 conical intersection. Therefore, ultrafast excited-state dynamics is expected to occur in the bright S_2 state, implying a highly efficient channel for internal conversion via this conical intersection.

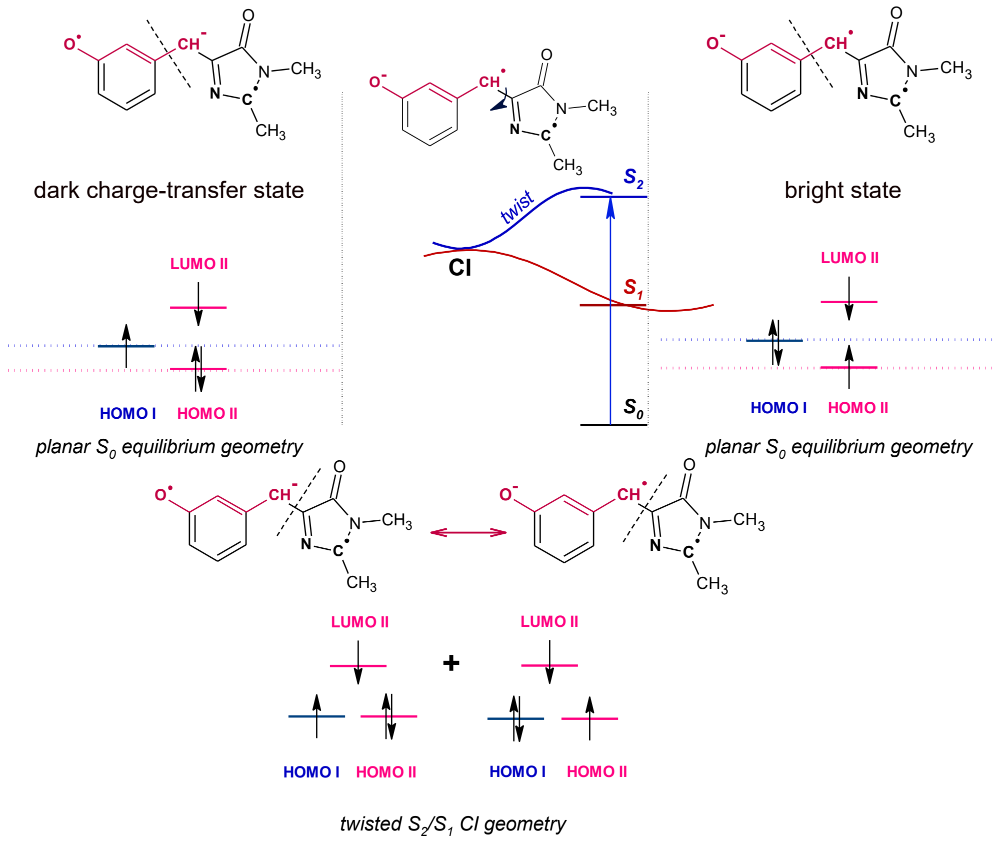

Figure S6: Simplified electronic structure of the wavefunction along the relaxation pathway through the twist of the bridge bond adjacent to the imidazolinone ring in the S_2 bright state. Two resonance structures exist at the geometry configuration of the conical intersection, which scramble excited bright and dark states.

Intramolecular rotation in S_2

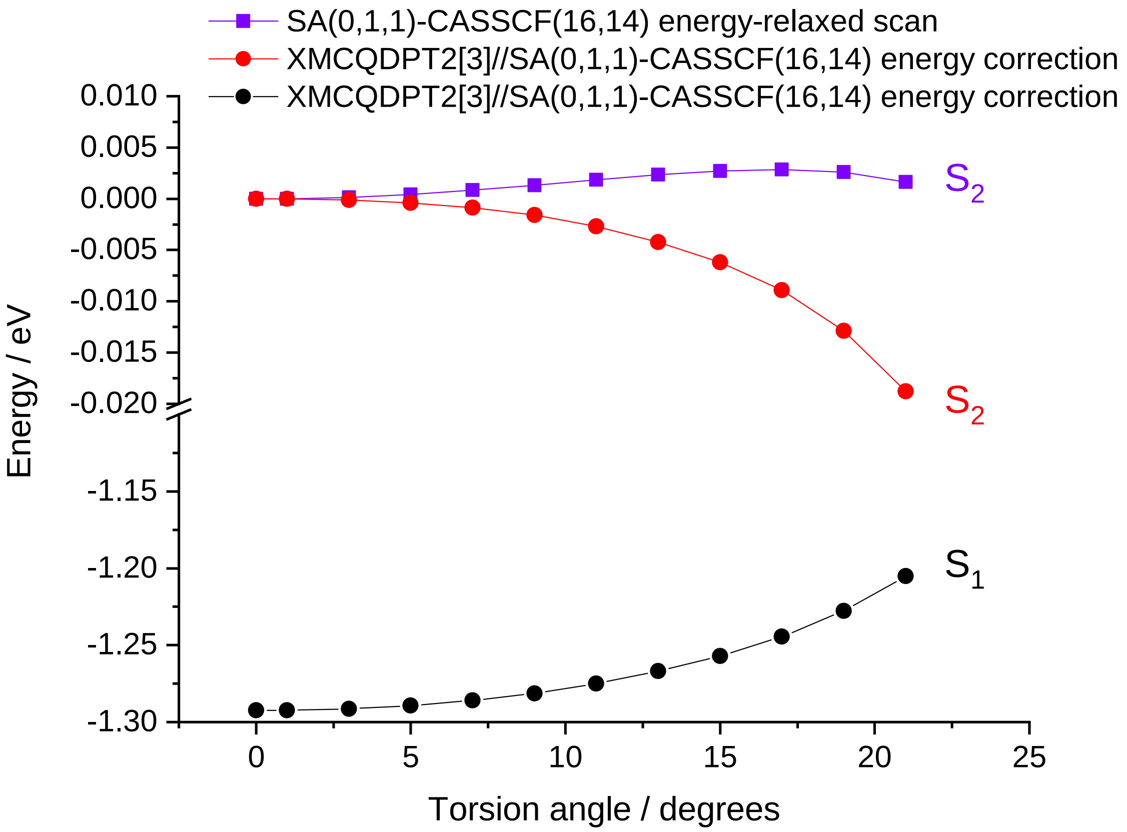

Figure S7: Calculated potential that hinders rotation about the double C=C bridge bond in meta-HBDI in the S_2 electronic state. The S_2 energy-relaxed scan along the C–C=C–N torsion coordinate is obtained at the SA(0,1,1)-CASSCF(16,14)/(aug)-cc-pVDZ level of theory, where the S_2 and S_1 states were included in the state-averaging procedure. The point energy corrections were calculated using the XMCQDPT2 [3]/SA(0,1,1)-CASSCF(16,14)/(aug)-cc-pVDZ method in the optimized geometries as a function of the C–C=C–N torsion angle. Note that there is no barrier in S_2 between the planar geometry, which corresponds to the Franck-Condon point, and the S_2/S_1 conical intersection reached by rotation about the double C=C bridge bond. The CASSCF potential is found to be more flat compared to that obtained using the XMCQDPT2 method. At the C–C=C–N angle of 17°, the CASSCF method yields a negligible barrier, which is less than 0.003 eV, while no barrier is observed at the XMCQDPT2 level of theory.

Conical intersections along the dark branch

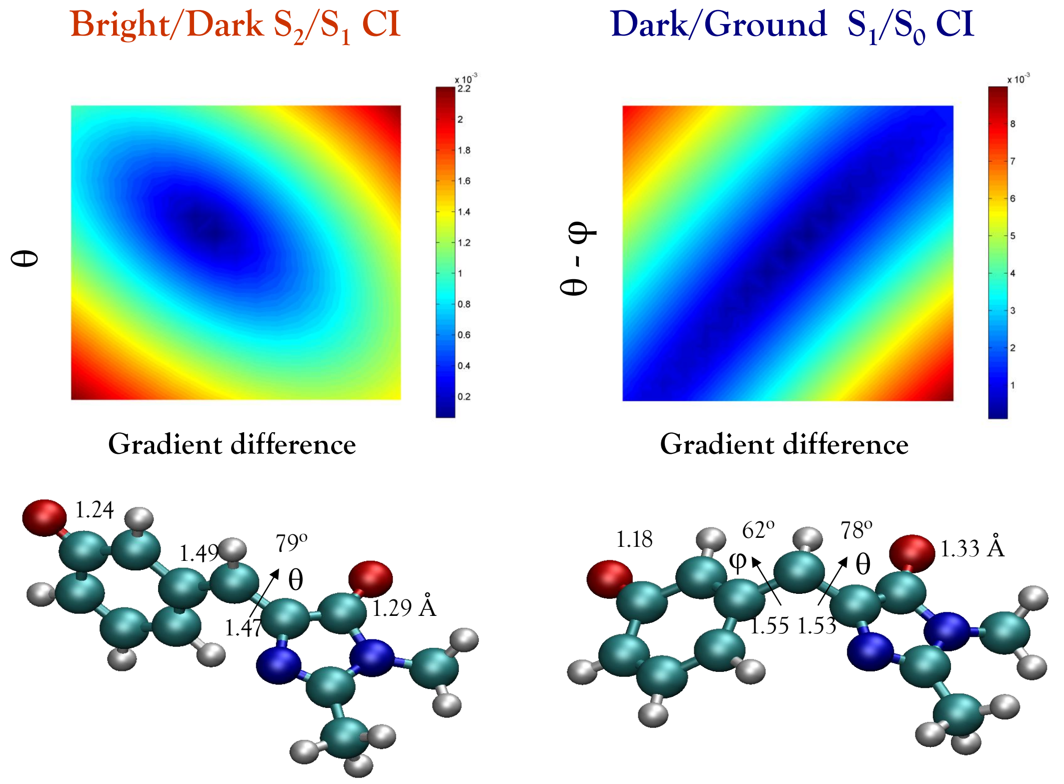

Figure S8: XMCQDPT2/SA(3)-CASSCF(14,13)/(aug)-cc-pVDZ minimum-energy structures of the S_2/S_1 and S_1/S_0 conical intersections (MECI) along the dark branch. Shown are also the topographies of the potential energy surfaces around the conical intersections plotted as the energy difference (in a.u.) between the two states in the corresponding branching planes. The branching planes are defined by two orthogonal coordinates: the nonadiabatic coupling vector, which corresponds to torsion of the bridge moiety (θ for the S_2/S_1 MECI and $\theta - \varphi$ for the S_1/S_0 MECI), and the gradient difference vector, which describes bond length alternation (BLA) in the conjugated π-system (see the accompanying animations in the SI). Note the peaked topography of the S_2/S_1 conical intersection and the sloped topography of the S_1/S_0 conical intersection, which are shown in Fig. 7 in the main text. The peaked MECI coincides with a local minimum in the upper S_2 state. In contrast, the sloped MECI is not a stationary point and lies higher in energy than the S_1 minimum. The structure of the S_1/S_0 MECI exhibits twisting about two bridge bonds and features significant piramidalization of the bridge carbon atom (43°). The associated energy barrier along the lowest-energy dark branch, which traps the population in S_1, is as large as 0.41 eV.

Calculated S_1 excited-state lifetimes

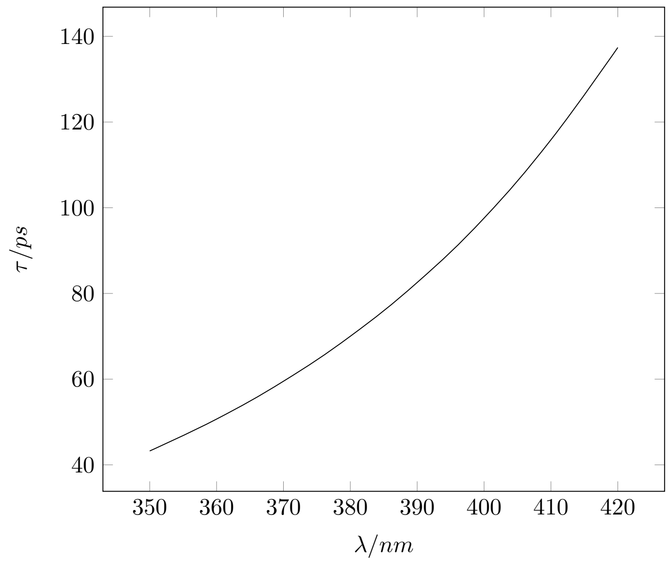

Figure S9: Calculated S_1 lifetime as a function of excitation wavelength at 300 K

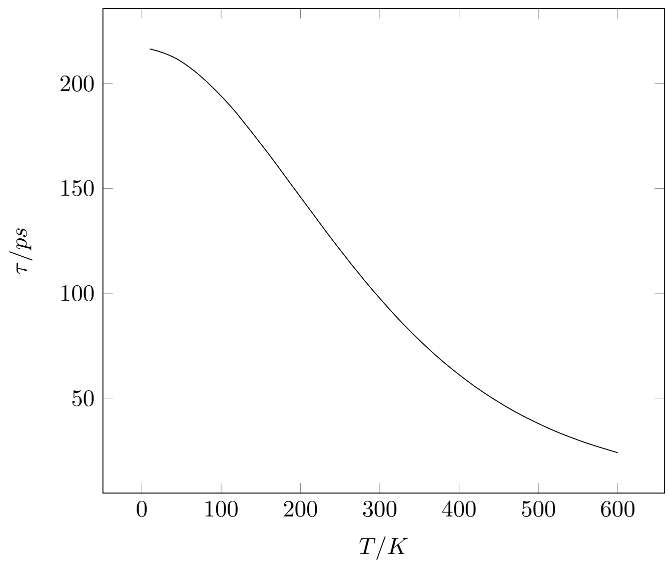

Figure S10: Calculated S_1 lifetime as a function of ground-state temperature under 400 nm excitation

References

(S1) Bochenkova, A. V.; Andersen, L. H. Ultrafast dual photoresponse of isolated biological chromophores: link to the photoinduced mode-specific non-adiabatic dynamics in proteins. *Faraday discussions* **2013**, *163*, 297–319.
