# Supplementary figures and images for: Dark-State-Mediated Efficient Energy Trapping in a Model GFP Chromophore
Source: J Am Chem Soc. 2026 Apr 17;148(16):16750–9. doi: 10.1021/jacs.5c22023 (PMC13133902; doi:10.1021/jacs.5c22023)

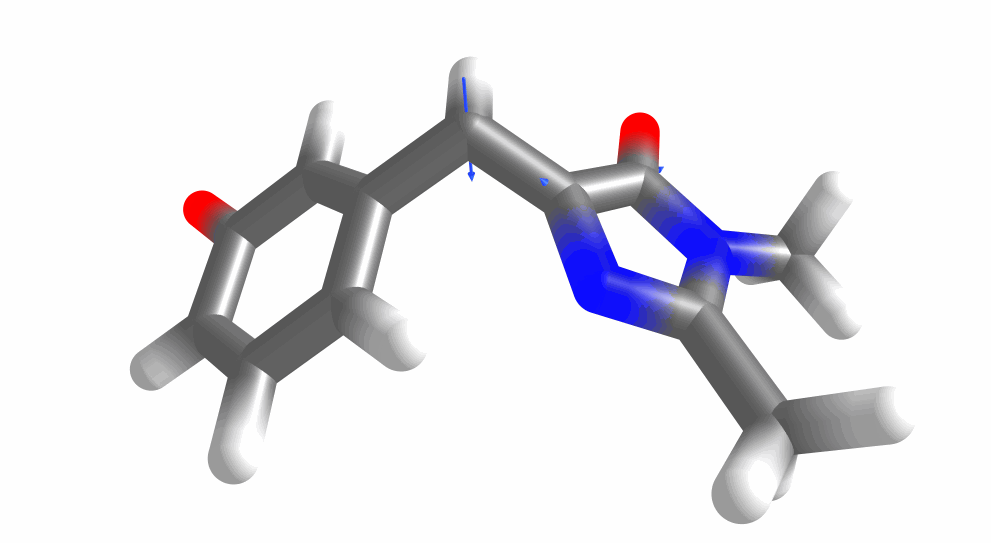

Supplement: Supplementary file 2 [file ja5c22023_si_002.gif]

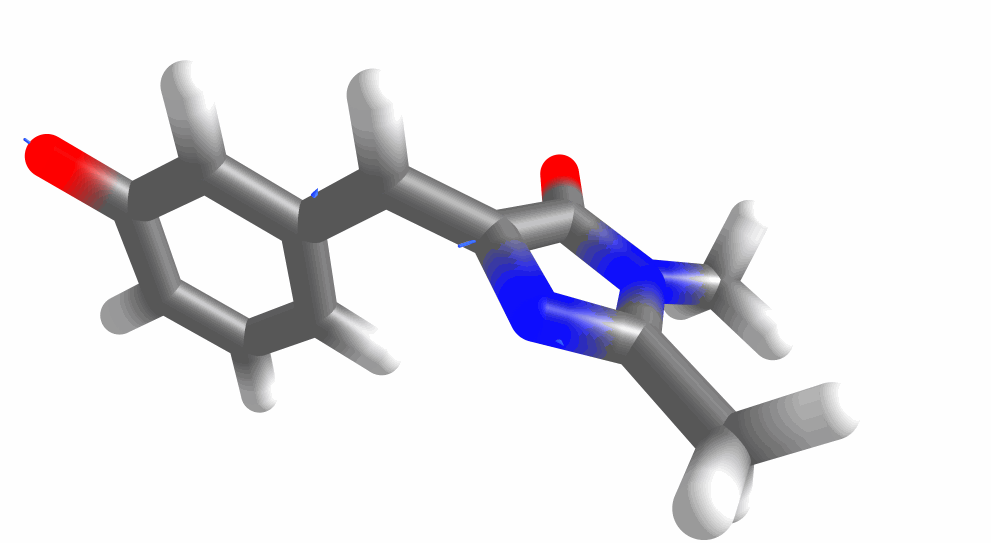

Supplement: Supplementary file 3 [file ja5c22023_si_003.gif]

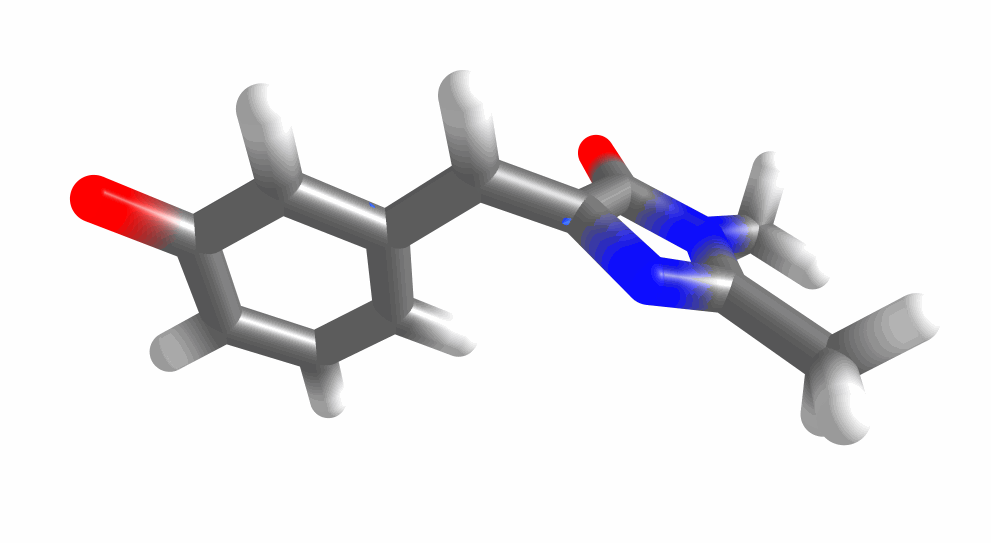

Supplement: Supplementary file 4 [file ja5c22023_si_004.gif]
